# Barriers and facilitators to oral PrEP uptake among high-risk men after HIV testing at workplaces in Uganda: a qualitative study

**DOI:** 10.1186/s12889-023-15260-3

**Published:** 2023-02-20

**Authors:** Racheal Nabunya, Victoria M. S. Karis, Lydia Joslyline Nakanwagi, Pius Mukisa, Patience A. Muwanguzi

**Affiliations:** 1 Department of Nursing, School of Health Sciences, College of Health Sciences, Makerere University, P. O. Box 7072, Kampala, Uganda; 2 Clinical Epidemiology Unit, School of Medicine, College of Health Sciences, Makerere University, P. O. Box 7072, Kampala, Uganda

**Keywords:** PrEP, High risk: men, Workplace HIV testing, Qualitative research

## Abstract

**Background:**

Men in Uganda contribute significantly to new HIV infections annually yet PrEP uptake among them is low and those initiated are likely to discontinue usage. We explored the barriers and facilitators to PrEP uptake among high-risk men employed in private security services with negative HIV results after testing at workplaces in Uganda.

**Methods:**

An explorative qualitative study comprising in-depth participant interviews. Data were collected via telephone calls and manually analyzed by inductive content analysis.

**Results:**

Fifty-six (56) men participated, 27(48.21%) had heard about PrEP, and 29(51.79%) were willing to initiate it. Four categories emerged for the facilitators of PrEP uptake including the perceived need for HIV prevention, awareness creation, availability, and sexual freedom. Six categories emerged for the barriers to PrEP uptake. These were: Inaccessibility of PrEP services, Misinformation, Knowledge deficit, Medication-related barriers, Potential for increased risky sexual behavior, and Perceptions about PrEP use.

**Conclusion:**

The findings suggest the need for healthcare providers to offer information regarding PrEP and HIV prevention services and mass sensitization campaigns to facilitate uptake. Participants recommend mass roll-out of PrEP to lower-level facilities and accessible pick-up points for men such as workplaces. The men also suggested the use of longer-acting PrEP modalities such as an injectable option or an option that is utilized specifically by the female partner. Finally, the stigma surrounding PrEP use could be reduced by the separation of PrEP and ART services at health facilities, or special pick-up days to reduce waiting times.

**Supplementary Information:**

The online version contains supplementary material available at 10.1186/s12889-023-15260-3.

## Background

Pre-exposure Prophylaxis (PrEP) is a course of antiretroviral drugs that HIV-negative people can take to prevent HIV acquisition [1]. The World Health Organization (WHO) proposed the use of PrEP for HIV-negative persons at high risk of infection including most at-risk populations (MARPs) [2]. PrEP is highly effective and reduces the risk of getting HIV through sex by 90% when taken as prescribed [3]. PrEP is taken by individuals at risk of getting HIV and one has to take it daily to prevent HIV infection through sex or injection drug use [4]. Eligibility for PrEP is likely to be more prevalent in populations at substantial risk of HIV such as discordant couples, sex workers, fisherfolk, long-distance truck drivers, men who have sex with men (MSM), uniformed forces, and adolescents and young women including pregnant and lactating adolescent girls and young women (AGYW) [5].

PrEP is being utilized globally with relatively high degrees of acceptability [6]. In the United States and the United Kingdom, PrEP has been well received and the desirability rate is good [7, 8]. Studies in Africa among men in Benin, [9] Nigeria, [10] and youth in Uganda, Zimbabwe, and South Africa have also shown high acceptability for PrEP [11]. In Uganda, PrEP was initially offered in a few accredited antiretroviral therapy (ART) sites with the capacity to provide a complete package. It has not yet been rolled out to all health facilities in the country [12, 13]. The Ugandan ministry of health (MoH) started the national PrEP rollout for HIV prevention in 2017 and this scale-up began with serodiscordant couples (SDC) and has since expanded to other MARPs [14]. The Uganda PrEP guidelines recommend that after meeting the substantial risk for HIV criteria, HIV-negative status should be confirmed using the national HTS algorithm. Additionally, one should test negative for Hepatitis B and have a creatinine test done before they initiate PrEP [5]. These tests are not always available at lower health facilities but rather at regional referral hospitals. This may be another reason why PrEP is not yet offered nationwide. Tenofovir disoproxil fumarate and Emtricitabine (TDF/FTC) are the approved drugs for use as PrEP, used in combination as a single pill [15].

Men contribute about 11,000 of the new HIV infections in Uganda and about 10,000 AIDS-related deaths among people above 15 years of age [16]. A study among men employed in security companies revealed that several of them have multiple sexual partners which may increase their risk of HIV [17]. Similarly, a study in southern Uganda among key and priority populations reported that PrEP uptake was low and those who were initiated were more likely to discontinue usage [18]. Facilitators and barriers to PrEP uptake have been established in prior studies globally, particularly among MARPS [12] including adolescents and young people [11, 19–21], MSM [22–24], transgender and gender non-binary people [25–28], fisherfolk [29], people who inject drugs [22], people in hospitals [30, 31], serodiscordant couples and truck drivers [32], sex workers [23], black communities [33, 34], and gay and bisexual men [35]. To our knowledge, there is limited documentation on the barriers and facilitators to PrEP uptake among men at substantial HIV risk who test HIV negative in work settings. Therefore, the study aimed at exploring the barriers and facilitators to PrEP uptake among high-risk men with negative HIV results after testing at workplaces in Uganda.

## Methods

### Aim

To explore the barriers and facilitators to oral PrEP uptake among high-risk men with HIV-negative results after testing at workplaces in Uganda.

### Study design and setting

The study was an explorative narrative study within the Workplace-Based HIV Self-testing among Men (WISe-Men) clinical trial. The protocol for the trial is presented elsewhere [36]. This was a two-arm cluster randomized trial (CRT) involving men employed in private security services in Uganda (clinicaltrials.gov registration number NCT04164433). The clusters were private security companies each employing more than 50 men. Data were collected from two Ugandan districts. In the experimental arm, men received HIV self-testing (HIVST), while in the control arm, men received HIV rapid diagnostic testing (RDT) according to the Ugandan ministry of health guidelines [5]. The data in this study were collected through telephone follow-up calls with trial participants within the first year of recruitment. The trial sought to determine the effect of HIV self-testing versus standard HIV testing services on the linkage to HIV care or HIV prevention services depending on the test outcome. As part of the trial, all the men who tested HIV negative were offered information about linkage to HIV prevention services. Information about PrEP was also given as many of the participants had never heard about it. Each participant received information on where to access the services and whom to contact if they had any difficulties. They also received an information packet with information about the HIV prevention services available in the nearby healthcare facilities. Information about barriers to the uptake of PrEP was collected from men who were not willing to initiate PrEP, while the data on the facilitators were collected from men who were willing to utilize PrEP. This qualitative study did not examine the contribution of the testing modality to the barriers and facilitators identified in this research.

### Participants

The participants in this study were men who received negative HIV test results and met the criteria for PrEP initiation. The trial participants were screened for the risk of acquiring HIV infection according to the Uganda national guidelines [5]. As part of the trial baseline information, any participant who responded “yes” to any of the following criteria was identified as being at high risk for HIV acquisition and consequently, eligible for participation in this study. These include (i) people who have had unprotected vaginal sexual intercourse with more than one partner of unknown HIV status in the past six months, (ii) have had anal sexual intercourse in the past six months, (iii) have had sex in exchange for money, goods or service in the last six months, (iv) use or abuse drugs especially injectable drugs in the last six months, (v) have had more than one episode of an STI within the last twelve months, (vi) are part of a discordant couple, especially if the HIV-positive partner is not on ART or has been on ART for less than six months or not virally suppressed. (vii) Recurrent post-exposure prophylaxis (PEP) users, (Recurrent implies PEP use more than 3 times a year) or (viii) are members of key or priority populations who are unable or unwilling to achieve consistent use of condoms [5].

### Data collection

Data were collected at the men’s workplaces. The employers granted permission to speak with the participants. Information sessions were held at the workplace and men who met the criteria and were interested in participation agreed on a convenient day and time for the interview. But due to COVID-19 restrictions, interviews were carried out through telephone calls [37]. Participants were purposefully sampled to include high-risk men with HIV-negative test results from different employee ranks, and age categories (19–30, 31–40, 41–50). Men at high-risk were selected based on the Ugandan Ministry of Health PrEP initiation guidelines. In-depth interviews (IDI) were conducted by RN and VMSK and two research assistants who were trained and have experience in qualitative methodologies. The interview guide was tested and developed iteratively at three pilot interviews to refine the questions. The study participants in the pilot interviews consented before participation. Enrolled study participants gave written informed consent to participate during the WISe-Men trial and further verbal consent was sought on phone before commencing the interview for this study. We collected data about participants’ views on the willingness, facilitators and barriers to PrEP uptake. Interviewers were in a private and quiet place and participants were asked to respond only if they too were in a quiet and private place. Of the 63 participants contacted, only 56 were interviewed giving a response rate of 88.89%. Three (3) participants declined participation while four (4) did not feel that the environment was conducive enough to participate. Audio files and transcriptions were safely stored on the study computer protected with a password only known to members of the study team to ensure confidentiality. The Phone IDIs were conducted from March through to November 2021 since participants for the clinical trial were recruited from February to December 2020. Each interview lasted 45 min to an hour. Participants were asked if they had ever heard about PrEP, their willingness to take it up and their reasons for their uptake or refusal.

### Data analysis

The interview recordings were transcribed verbatim daily. The data from study participants were analyzed manually using inductive content analysis. This process entailed open coding, developing emergent categories, and conceptualization [38]. Initially, RN and VMSK reviewed the transcripts independently. They each identified codes and discussed them to reach a consensus. Any disagreements about the codes were settled by discussions with other members of the study team. As more insights and latent meanings emerged from the data, the coders iteratively named and re-named the codes which were then grouped into subcategories and categories.

To ensure the trustworthiness and credibility of the data, a sample of the study participants reviewed the categories and subcategories. The sample (n = 4) included two participants from each testing group. The reviewers read through the identified categories and sub-categories to validate them as a true representation of their perspectives of barriers and facilitators to PrEP uptake. The participants contributed to all categories and sub-categories. Interview notes were recorded in the principal researcher’s reflective journal for confirmability.

## Results

We interviewed 56 participants; 28 received negative results after HIVST and 28 after HIV RDT. Most of the participants were 19–30 years with a mean of 29.07(SD 7.99) and 78.5% had attained post-secondary education. The mean number of sexual partners in the last 12 months was 4(SD 1.31). Twenty-seven participants (48.21%) had heard about PrEP, while 29(51.79%) were willing to initiate it. Two men (3.57%) had sex with a male partner in the last six months although they do not self-identify as gay (Table 1).

**Table 1:** Demographics of participants

Characteristics	HIVST group (n = 28)	HIV RDT group (n = 28)	Combined groups (N = 56)
**Age**			
Mean(SD)	30.93(8.12)	27.21(7.86)	29.07(7.99)
< 19 years	1	1	2
19–30 years	14	18	32
31–40 years	10	8	18
41–50 years	3	1	4
**Education level**			
No formal schooling	1(3.57%)	1(3.57%)	2(3.57%)
Secondary	2(7.14%)	1(3.57%)	3(5.36%)
Tertiary	21(75%)	23(82.14%)	44(78.57%)
Completed tertiary	4(14.29%)	3(10.72%)	7(12.5%)
**Marital status**			
Married/living as married	15(53.57%)	18(64.29%)	33(58.93%)
Single	13(46.43%)	8(28.57%)	21(37.50%)
Separated/divorced	0	2(7.14%)	2(3.57%)
**Number of sexual partners (past 12 months)**			
Mean (SD)	3.96(1.14)	4.18(1.47)	4(1.31)
2	0	2	2
3	11	10	21
4	11	4	15
5	4	8	12
> 5	2	4	6
**Sex with a male partner (past 6 months)**			
Yes	0	2(7.14%)	2(3.57%)
No	28(100%)	26(92.86%)	54(96.43%)
**Ever heard about PREP**			
Yes	12(42.86%)	15(53.57%)	27(48.21%)
No	16(57.14%)	13(46.43%)	29(51.79%)
**Willing to initiate PREP**			
Yes	13(46.43%)	16(57.14%)	29(51.79%)
No	15(53.57%)	12(46.43%)	27(48.21%)

## Barriers and facilitators to PrEP uptake

The findings are presented under two main sections: barriers and facilitators.

## Facilitators to PrEP uptake

**Fig. 1:**
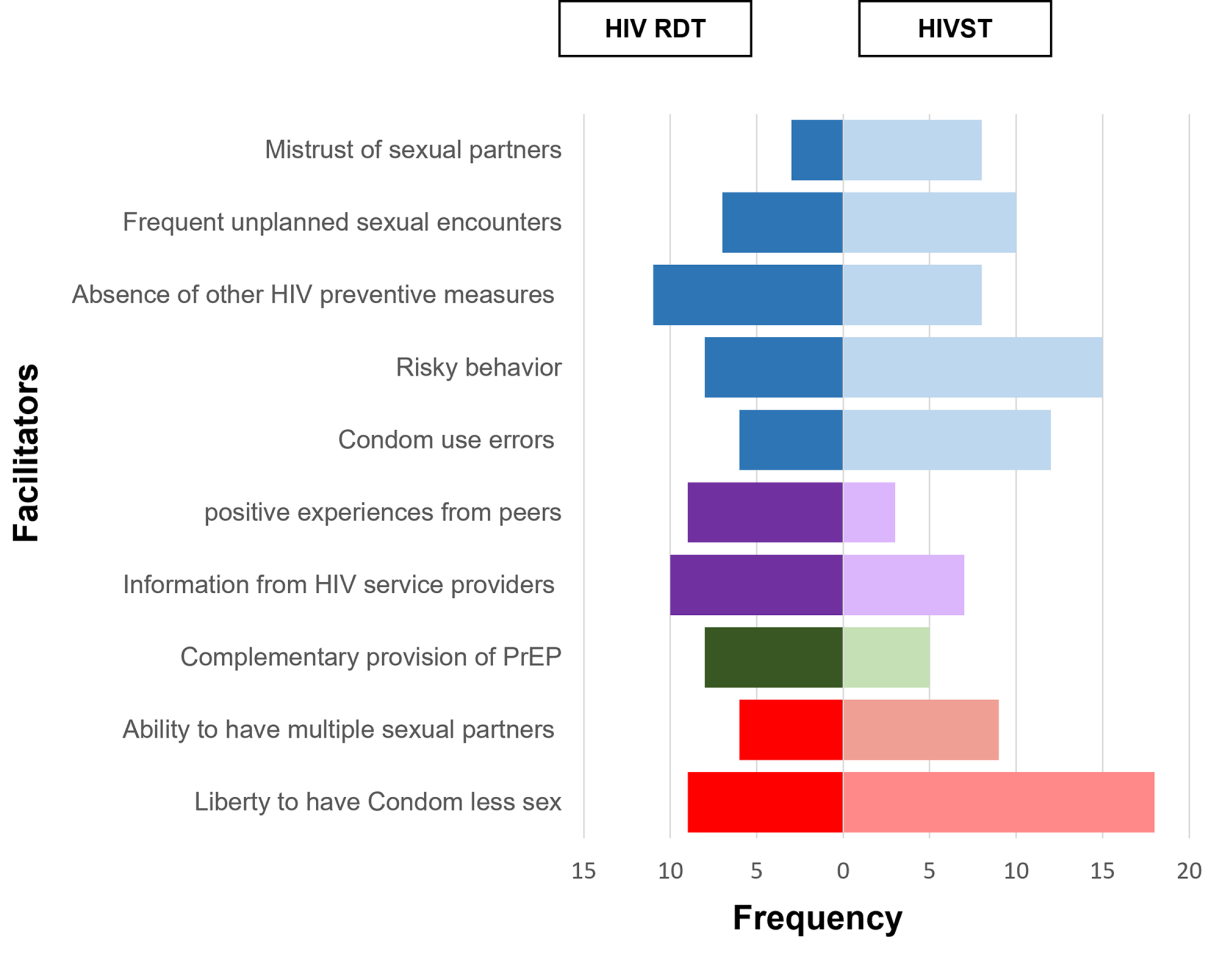
Frequency of mentions for facilitators of PrEP uptake

Four main categories emerged from the analysis, perceived need for HIV prevention, awareness creation, availability, and sexual freedom. Figure 1 presents the frequencies of mentions of facilitators to PrEP uptake. The subcategories and narrative quotes are presented in supplementary Table 1.

## Perceived need for HIV prevention

***Mistrust of sexual partners.*** This arose as one of the major reasons for PrEP uptake. In Uganda, men in security services experience high mobility, often spending long periods of separation from their families. Many men seemed unsure about their partners’ sexual activities since they worked far from home most of the time and were unable to monitor their partners’ engagements at the time of separation. Therefore, they felt that PrEP would help to prevent HIV acquisition from their sexual partners when they returned home from their long periods away. When asked about condom use, these participants were unwilling to use condoms with their long-term sexual partners.

***Frequent unplanned sexual encounters.*** Because of their nature of work as security guards, they typically work irregular and arduous shifts, while others are deployed to other districts away from their families. Other men leave their families settled in larger or extended communities in rural areas and relocate to towns or cities for this work. Therefore, they sometimes experience unplanned sexual engagements without prior preparation, limited information about the sexual partner, and protection like condoms, which greatly exposes them to infection risk. Consequently, the participants felt that utilizing PrEP could be beneficial in the event of these unplanned sexual engagements. They verbalized that it would help them to be prepared and protected at any time.

***Absence of other HIV preventive measures.*** Some participants reported that they are frequently posted to offer security services in hard-to-reach or rural areas where HIV prevention measures like condoms or SMC (safe male circumcision) may not be readily available. Therefore, they felt that if they could receive PrEP, they would go to their work placements confident of their protection against HIV acquisition. Those in the cities reported that they could not afford to buy some of the preferred measures such as condoms that may be available in private hospitals, pharmacies, and clinics.

***Risky behavior.*** Participants were asked about their risk of HIV acquisition, and several perceived themselves as low-risk. They believed that it was not harmful to an individual to have occasional sexual encounters with a partner whose HIV status they did not know. They also reported inconsistent condom use which may put them at low risk for HIV acquisition. However, HIV risk was not objectively measured for this study, therefore the responses are based on their perceived HIV risk. Some participants who self-identified as medium to high risk were willing to take up PrEP to feel protected. This is because they have unprotected sex often with multiple casual sexual partners including sex workers or random sexual engagements and later worry about the consequences. Some participants were surprised that they returned negative HIV test results and were willing to initiate PrEP since they felt that it could protect them against HIV given their risky sexual behavior.

***Condom use errors.*** Some respondents had experienced challenges using other prevention methods like condoms. They reported condom bursts, rolling off, difficulty with finding appropriate sizes, and wrong use during sexual encounters. They acknowledged that this puts people at risk and can result in new infections therefore to avoid this, some participants were willing to start PrEP. The men contended that if one adhered to their medication, there was less likelihood of errors, such as those previously experienced with condoms.

## Awareness creation

Sensitization of the public about PrEP from trusted sources is a critical driver for PrEP uptake.

***Correct Information and positive experiences from peers.*** Family and friends were key to the participants’ willingness to initiate PrEP. Several participants reported that they were only willing to start PrEP after hearing about positive experiences from their peers and work colleagues. The success stories and messages shared were a source of reliable and trusted information, especially from peers. Other participants had read about PrEP experiences from social media platforms such as Facebook and WhatsApp, however, when they discussed with their colleagues who initiated PrEP, they realized that some of the information they received was incorrect. Therefore, they recommended public sensitization regarding PrEP to debunk several existing myths and misinformation.

***Information from HIV service providers.*** Approximately 29(51.79%) participants had never heard about PrEP, while others had incorrect information about it. However, some participants were willing to start PrEP because of the information they received through media and community outreach drives, counselling, and guidance from healthcare providers. The men reported that they trust the information they receive from healthcare workers regarding HIV prevention, therefore correct information provided by trained health providers was a motivator for PrEP uptake.

## Availability

***Complimentary provision of PrEP.*** Affordability was a great motivator for PrEP initiation. The participants were concerned about the cost since it is a medication taken for the time when one is at risk, and they felt that they were constantly at risk most of the time. Most respondents reported the inability to purchase from pharmacies, private hospitals, and clinics therefore accessing PrEP freely from health centers, hospitals and all clinics in the community makes it easier for people to choose PrEP as a preventive measure. The participants even suggested the delivery of PrEP to their workplaces.

## Sexual freedom

***Ability to sexually engage with multiple sexual partners***. One motivator for PrEP uptake was the freedom to have multiple sexual engagements. The participants found PrEP appealing because it could potentially allow them to have multiple casual sexual partners without necessarily being attached to them or worrying about acquiring HIV. PrEP is therefore attractive to some of the respondents who want a free sexual life. We discussed with the participants that while PrEP is prevention for HIV, it does not prevent the acquisition of other STIs. The men expressed more concern about HIV because they felt that the other STIs were treatable.

***Liberty to have condomless sex.*** Some of the respondents felt that condoms limit their enjoyment of sex, and they expressed a preference for sexual activities without a barrier. Unfortunately, this is not possible when one has multiple sexual partners, therefore the need for protection outweighs their pleasure and they typically use condoms. The men verbalized that PrEP was alluring because it allows one to exercise their right to sexual enjoyment while affording them protection against HIV infection.

## Barriers to PrEP uptake

**Fig. 2:**
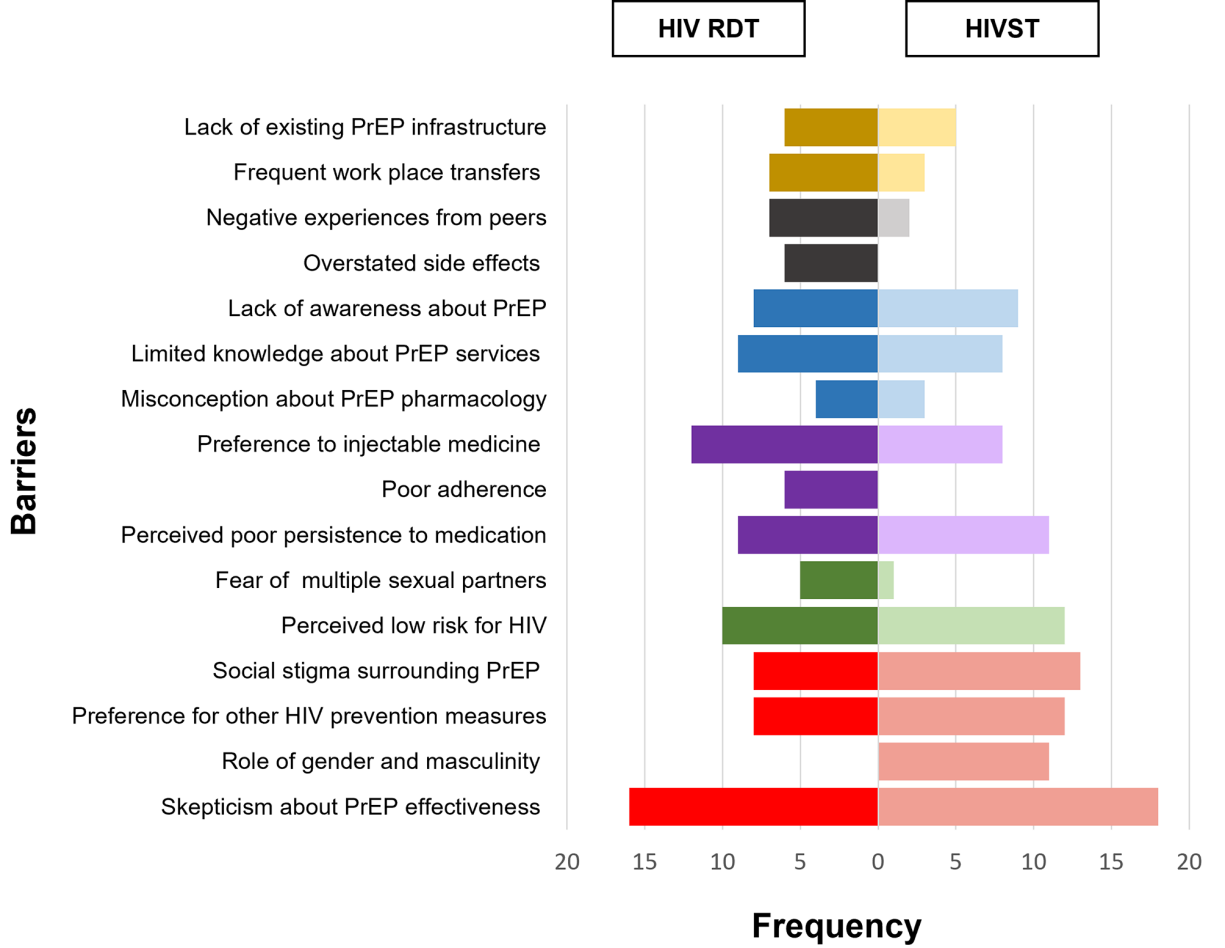
Frequency of the mentions of barriers to PrEP uptake

Six categories emerged, Inaccessibility of PrEP services, Misinformation, Knowledge deficit, Medication-related barriers, Potential for increased risky sexual behavior, and Perceptions about PrEP use. Figure 2 presents the frequencies of the mentions of barriers to PrEP uptake. The subcategories and narrative quotes are presented in supplementary Table 2.

## Inaccessibility of PrEP services

***Lack of existing PrEP infrastructure at lowest health facilities.*** The men decried the absence of PrEP at the lower health facilities since it has not yet been rolled out to all levels of the healthcare system. They remarked on the lack of PrEP systems and infrastructure and medications. For example, one participant who was ready to be initiated on PrEP reported that he could not access the services when he was transferred from an urban to a rural location. This was a major barrier for many because they felt that such services should be conveniently located if they want people to use them.

***Frequent workplace transfers.*** All the participants were men employed in private security services as security guards. The nature of their work is not permanent; therefore, they are frequently transferred from one work site to another. Occasionally, these may be in a different area or district. Due to the different inequalities in the health sector, the transferred workers may not find conducive support to continue PrEP uptake in the new places. This creates disruptions and poor adherence to the drug and hence low or zero effectiveness. For example, two participants reported that they were transferred, and the closest health facility did not have PrEP services, thus, they could not access the medications until they returned to their office headquarters. Therefore, frequent work transfers were an enormous barrier to PrEP uptake.

## Misinformation

***Negative experiences from peers.*** When the participants heard about PrEP, they consulted peers who mainly shared negative experiences and the challenges they faced since starting PrEP. The negative experiences included information such as the stigma that their peers faced. The men reported that people who knew that they were taking PrEP thought that they were HIV positive or MSM since many people think that PrEP is for such populations. These experiences were key in shaping their decision not to initiate PrEP. The negativity planted fear and worry in the minds of the would-be PrEP users and hindered their objective decision for PrEP utilization. The participants recommended using peers as a potential avenue to share correct information and positively influence their colleagues to take PrEP since they trust their word.

***Side effects.*** Another barrier to PrEP uptake was the fear of getting side effects that were similar to or worse than those experienced by their colleagues. Negatively stated side effects by people who had used PrEP and ARVs created a scare among the men who tested HIV-negative. In some cases, the work colleagues and friends were not sharing their own experiences but secondary information that they had picked up from other people. This may have resulted in changes in the information leading to misinformation and exaggerated incidence and severity of side effects.

## Knowledge deficit

***Lack of awareness about PrEP.*** About half of the respondents 29(51.79%) had never heard about PrEP and were not aware that it exists. However, following discussions with the participants and information sessions, some were able to make an informed decision to take up PrEP. This highlights the importance of sensitization of the public regarding health initiatives.

***Limited knowledge of PrEP services.*** While some participants had heard about PrEP, many were not sure where to seek PrEP services. They did not know whether one was given PrEP for the whole year, given at any general hospital, or whether they could receive it in the community or obtain it from an HIV clinic. They suggested that this limited knowledge was a barrier to uptake because they did not even know where to go and inquire about PrEP.

***Misconception about PrEP pharmacology.*** Misconceptions especially among those with limited knowledge about PrEP deterred many from accepting PrEP. They needed to be sure of how the drug works, its effectiveness, side effects, and its expected outcomes before usage. Others had a wrong perception that PrEP could prevent other viral infections besides HIV. The participants were also concerned about using ART for prevention and needed specific information on the difference between PrEP and ARVs.

## Medication-related barriers

***Preference to injectable medicine.*** Some respondents expressed a preference for injectables over oral PrEP. They did not like the idea of swallowing medication daily. They suggested a PrEP injection that could be taken once a month or every three to six months. They verbalized that they were more likely to remember one date every six months than a daily pill.

***Poor adherence.*** Difficulty sticking to prescribed medication was expressed as an issue for most of the would-be PrEP users. They reported challenges with completing other routinely prescribed medication doses. Therefore, this discouraged them from uptake for fear of inconsistency. They were concerned about remembering to take the medication, especially given the grueling nature of their work. Other respondents remarked that their wives usually assist with pill reminders. However, since their partners were far, they could not be sure to remember on their own to take the medications daily. Participants with already existing chronic medical conditions like hypertension and diabetes felt that adding another pill to the already existing daily medications could potentially increase the pill burden.

***Perceived poor persistence to medication.*** Several respondents were willing to take PrEP and felt that they could adhere to the regimen. However, they expressed concern about commitment to taking the medication for long periods. Some participants seemed to prefer a quick-fix solution like condoms or taking treatment for a short period like in the case of post-exposure prophylaxis (PEP) but not for life.

## Potential for increased risky sexual behavior

***Fear of engagement with multiple sexual partners.*** Some respondents were not willing to take PrEP because they felt that this assurance of protection might lead them to careless sexual engagements. For example, they might get involved with an individual whose status is not known or with several partners. They were concerned that PrEP uptake may damage their values and corrupt their religious morals of sticking to one partner.

***Perceived protection from HIV.*** Some respondents have misconceptions that their immune systems are strong enough and they cannot easily get infections including HIV. Some of these people have never had any serious illness and therefore think that they cannot easily acquire HIV. The participants felt that having a good general health status gave them immunity from all other illnesses including HIV. Therefore, they did not see the need to utilize PrEP since they had a strong innate immune system.

## Perceptions about PrEP use

***The social stigma surrounding PrEP.*** The respondents decried the existing social stigma surrounding PrEP users. They indicated that PrEP was associated with sex workers and gay men. They did not want to risk being alienated and stigmatized therefore they were not willing to take up PrEP. In other instances, the men did not want to take PrEP because they were concerned that people might think that they were taking ARVs and had HIV. They, therefore, recommended sensitization of the public regarding the difference between PrEP and ART, and the separation of PrEP and HIV services at health facilities.

***Preference for other HIV preventive measures.*** There was a preference for other HIV preventive measures and campaigns. Many of them cited the campaign from 1991 dubbed ‘ABC’ *Abstinence, Be faithful and Condom use* which was championed by the first lady of the country, and the mass sensitization made it memorable [39]. They also preferred measures that they could control and did not enter their bloodstream. The respondents suggested that it might be beneficial to offer each person several options so that they select the method they are most comfortable with. This ensures that no matter which option they choose, people are utilizing HIV prevention measures.

***Role of gender and masculinity.*** Some of the men were concerned about starting PrEP individually and then having to explain this to their partners. Therefore, this sub-category surfaced in terms of the need to consult with a sexual partner to seek consent or get their opinion concerning HIV prevention measures. This recurred especially among those that work away from home. Starting PrEP as an individual may breed mistrust in the relationship. Therefore, this caused delays in uptake and refusal in case the sexual partner did not want them to use PrEP. In other instances, the men felt that this was a barrier because they needed to discuss this with their female partners, and some felt like this defied the gender roles in the home.

***Skepticism about PrEP effectiveness.*** Respondents lacked confidence in the effectiveness of PrEP in protecting them against HIV. They were concerned that HIV has no cure and thus wondered how the same medication could prevent its acquisition. They had also not heard testimonies and stories from people who were using PrEP and it had worked for them. This further strengthened their unbelief in the efficacy of PrEP.

## Discussion

This study aimed to explore the barriers and facilitators to PrEP uptake among high-risk men with negative HIV test results after workplace-based testing. Four categories emerged for the facilitators to PrEP uptake including perceived need for HIV prevention, awareness creation, availability, and sexual freedom. Six categories emerged for the barriers to PrEP utilization, Inaccessibility of PrEP services, Misinformation, Knowledge deficit, Medication-related barriers, Potential for increased risky sexual behavior, and Perceptions about PrEP use.

Several participants expressed willingness for PrEP uptake because of a perceived need for HIV prevention since they had tested HIV negative. This agrees with reports that men who consider themselves to be at risk of contracting HIV have shown a high uptake for PrEP [40]. Previous studies among this population suggest that they fear taking an HIV test because of their sexual behavior and fear of the unknown [41]. Furthermore, self-perceived risk and risky sexual behaviors are also considered one of the drivers of PrEP uptake in the United States [8, 42]. A study in Nigeria also suggested that the primary reason for accepting PrEP was because it provided an additional level of protection against HIV [43]. Therefore, high-risk men may be willing to utilize PrEP because HIV prevention is a major concern given the risky sexual behavior of multiple sexual partners.

Correct information about PrEP was a critical facilitator for PrEP uptake. Half of the participants had never heard about PrEP but when explanations were provided, several were willing to utilize it. The participants recommended that correct information about PrEP, including its side effects and efficacy should be given to the public from a trained healthcare provider since many discovered that they received the wrong information from their peers. Previous studies suggest that many men are willing to utilize PrEP if they access correct information [9]. Our findings are also in line with a study that showed low PrEP awareness but a high willingness to its use once information about PrEP was provided to them [43]. Findings from a study in Brazil expressed the need for awareness creation about the relevance and extra advantages of using PrEP to increase uptake [25]. Information about side effects was crucial in decision-making regarding PrEP uptake. This raises the need for PrEP awareness campaigns on various communication and media platforms, including social media.

Several study participants were willing to utilize PrEP if it was provided at no cost. Concerns about cost and affordability were raised and the participants advised that since it is a medication taken as long as one is at risk, it should be provided free of charge as many of them do not earn enough to afford the daily medication. This agrees with findings from Israel where willingness to use PrEP could be increased if structural barriers such as stigma and paying for PrEP were removed [44]. Similarly, not having to pay for PrEP was one of the facilitators to its uptake among men in Benin, [9] while in the US, cost and access were not seen as barriers [45]. Although in another study in the US, requests for medication cost assistance programs and discounts on PrEP medication were made by gay and bisexual men (GBM) [46]. The provision of PrEP free of charge was shown to increase its uptake with just a few of the participants willing to pay for it [47]. In this study, the men suggested PrEP availability at the workplace as a way of improving accessibility. In Nigeria, better awareness and correct information were shown in states where PrEP was available [48]. Providing PrEP along with other health services may increase its uptake at health facilities.

One of the drivers for the uptake of PrEP was that participants felt that PrEP gives one the chance to engage in multiple sexual relations because of perceived protection from HIV. They believe that PrEP bestows a level of protection against HIV and lowers the risk of transmission. Similarly in India, one of the drivers for PrEP uptake was so that men can have sex without condoms [49]. Additionally, it was also to increase sexual pleasure, because men were having sex without anxiety with various partners because they were assured of protection [49]. In Benin, the opportunity to have condomless sex with HIV-positive individuals came up as a good reason for taking PrEP [9]. Likewise, a study in Kenya and Uganda also reported that youth envision PrEP as a means for safely pursuing multiple partners [50]. On the other hand, low HIV risk perception among people has been identified as a barrier to PrEP uptake rather than a facilitator [8, 40]. Therefore, there is a need for men to understand their level of risk for HIV and for healthcare providers to offer information about PrEP related to that risk.

One major barrier to PrEP uptake was the challenge of lack of access to the services. Several participants decried the lack of PrEP services at the lowest health facilities, yet they receive frequent work transfers, sometimes to remote locations where the nearest health facilities do not offer PrEP services. In a study in the US, suburban MSM residing in PrEP deserts (defined as > 30-minute drive to access PrEP) were less likely to use PrEP than suburban MSM not residing in PrEP deserts [51]. Similarly, a study carried out in Uganda, Zimbabwe and South Africa reported unavailability due to drug stockouts at health facilities as one of the potential barriers to PrEP uptake [11]. Long distance from the PrEP clinic was one of the reasons given by men in South Africa [23] and likewise, a study in Kenya among fishermen highlighted walking long distances to dispensing facilities as a barrier [52]. The participants in our study recommended that PrEP be scaled-up even to the lowest health facilities for easy accessibility. This highlights the urgent need for PrEP scale-up across all levels of the health system in Uganda, particularly in rural areas.

Misinformation about PrEP, its efficacy, and side effects coupled with negative experiences of ART use from peers emerged as some of the reasons why some men were not willing to take PrEP. This agrees with the findings that one of the main reasons for unwillingness to take PrEP was the fear of side effects [53]. The participants recommended that correct information about PrEP, its efficacy, and side effects should be availed to the public so that men receive the right information to enable them to make an informed decision to use PrEP. This was also recommended because many of the participants were naïve about PrEP and had never even heard about it. Similarly, participants from a study in Uganda, Zimbabwe, and South Africa reported that information from friends and peers led to many misconceptions, yet it was their primary source of PrEP knowledge [11]. This was a similar finding among men in China where the PrEP awareness rate was low [47]. The men proposed that mass sensitization campaigns about PrEP should be carried out by trained HIV service providers to address issues of doubt and rumors from peers and friends. Information about advantages, effectiveness, side effects, and differences from ARVs should specifically be addressed during the sensitization. This agrees with a study where MSM perceived PrEP information rarely diffused in their rural, local communities, and more available on online sites, thus, they recommended sensitization via regularly used media such as radio and television [54].

The idea of taking pills daily for a long period was a key barrier to the uptake of PrEP for several participants. This was similar to findings from China, where one of the main reasons for unwillingness to take PrEP was the requirement for medication persistence and adherence [53]. Participants preferred long-acting measures like injectable intermittent PrEP that one can take for several months [47]. Participants with pre-existing health conditions found it hard to add PrEP to their repertoire of pills as they felt that this may worsen the existing pill burden. They recommended an easier means of taking PrEP such as a long-acting injectable or PrEP for female partners as this also helps with adherence. Findings from a study in South Africa additionally report that only 11.1% of the study MSM were willing to adhere to PrEP, [23] Similarly in Zimbabwe fear of the pill burden and pill impact on the general body emerged as a barrier to PrEP uptake [55]. This finding concurs with a study where adding another pill (PrEP) was seen as prohibitive for individuals with chaotic lives or those who were already taking other medications [26].

The stigma that surrounds ARVs and the belief that they are taken by only HIV-positive individuals made many shy away from PrEP. Additionally, the insinuation that PrEP is for MSM and people at high risk of HIV was a barrier to uptake. This is similar to findings where PrEP is seen as a ‘gay’ drug [56]. Taking daily tablets is typically related to people living with HIV which can be mistaken for treatment rather than prevention [11]. Several participants in this study were unwilling to take PrEP because they preferred non-biomedical forms of HIV prevention. Many cited previous campaigns in Uganda such as the ”Abstinence, Be faithful and or Condom Use” (ABC) approach because it was easier to practice and had no side effects. Additionally, other campaigns and HIV prevention methods do not raise questions from partners and are not linked to risky sexual behavior [9]. The stigma around PrEP use also makes it hard for one to disclose to their partner as this makes the partner assume that they are putting themselves at risk of HIV [49]. Therefore, our findings suggest that issues surrounding PrEP use stigma need to be thoroughly addressed to increase its uptake. The participants also recommended that in addition to other HIV preventive measures, PrEP should be accessed at special pick-up points, like their workplaces so that they can easily access the services. This was both as a stigma reduction strategy and to minimize costs and inconveniences associated with seeking HIV prevention services at health facilities. A suggestion of special clinic pick-up days was also made so that they do not spend a lot of time in lines and queues waiting for PrEP. Additionally, because of the stigma surrounding HIV, the participants suggested that the PrEP clinic be separated from the general HIV clinic as this gives them confidence, and people do not wrongly assume that they are accessing ART.

This is the first study to provide information about barriers and facilitators to PrEP uptake among men who have received HIV-negative results after workplace testing and who are at substantial risk of HIV acquisition. Data were collected as part of a clinical trial from participants who received negative HIV results following HIVST or HIVRDT at their workplaces. The findings from this study may be relevant to similar settings. The limitation of this study is that the use of telephone interviews made it difficult to read non-verbal cues during the interviews. An additional limitation is that we assessed the barriers and facilitators to men’s willingness to initiate PrEP but did not assess their uptake of PrEP.

## Conclusion

The study aimed to explore the barriers and facilitators to PrEP uptake among men with multiple sexual partners who tested negative for HIV in work settings in Uganda. Four categories emerged for the facilitators to PrEP uptake, and six for the barriers. We make the following recommendations from the study’s findings. First, there is a need for healthcare providers to provide information regarding PrEP including advantages, effectiveness, side effects, and difference from ARVs since many participants made their decision to utilize PrEP based on this information. Additionally, we recommend mass sensitization campaigns to facilitate uptake. Second, issues of lack of access and unavailability of PrEP emerged as key barriers to uptake, therefore we suggest rolling out to lower-level facilities and accessible pick-up points for men such as workplaces. Third, we suggest that PrEP services continue to be offered at no cost to enable uptake. Fourth, the men were concerned about adherence and persistence to PrEP therefore, they suggested longer-acting PrEP such as an injectable option or an option that is utilized specifically by the female partner. Finally, the findings show that there is a stigma surrounding PrEP use because it is normally recommended for high-risk men and the daily pill use may be misinterpreted as ART. Therefore, the participants recommend the separation of PrEP and ART services at health facilities, or special pick-up days to reduce waiting times.

## Electronic supplementary material

Below is the link to the electronic supplementary material.

Supplementary Material 1

Supplementary Material 2

## Data Availability

The datasets generated and/or analyzed during the current study are not publicly available to protect the privacy of study participants but are available from the corresponding author upon reasonable request.

## Abbreviations

ABC: Abstain, Be faithful and or Condom use.
AGYW: Adolescent Girls and Young Women.
ART: Antiretroviral Therapy.
ARV: Antiretroviral.
CRT: Cluster Randomized Trial.
FTC: Emtricitabine.
GBM: Gay and Bisexual Men.
HIVST: HIV Self-testing.
IDI: In-depth interview.
IRB: Institutional Review Board.
MARPS: Most At-Risk Populations.
MoH: Ministry of Health.
MSM: Men who have sex with men.
PEP: Post-Exposure Prophylaxis.
PI: Principal Investigator.
PLWHA: people living with HIV/AIDS.
PrEP: Pre-Exposure Prophylaxis.
RDT: Rapid Diagnostic Testing.
TDF: Tenofovir Disoproxil Fumarate.
UNAIDS: Joint United Nations Programme on HIV/AIDS.
UNCST: Uganda National Council for Science and Technology.
WHO: World Health Organization.
