# Supplementary material for: Barriers and facilitators to oral PrEP uptake among high-risk men after HIV testing at workplaces in Uganda: a qualitative study
Source: BMC Public Health. 2023 Feb 20;23:365. doi: 10.1186/s12889-023-15260-3 (PMC9940677; doi:10.1186/s12889-023-15260-3)
Supplement: Supplementary file 1 — Supplementary Material 1 [file 12889_2023_15260_MOESM1_ESM.docx]

**Supplementary table 1: Subcategories and narrative quotes for facilitators to PrEP uptake**

| Subcategory | Narrative quotes |  |
| --- | --- | --- |
| Mistrust of sexual partners | *“I love my life and with the work I do, sometimes you are away from home for so long and you don’t know what the woman is doing in your absence. So, if I am taking PrEP which I have taken before, it helps to protect you in case the woman is not faithful. Like me, I took it because I was not trusting my woman.”* (Participant 25, HIVST)  *“For as long as it is available because in most times, we security people are far from our partners and when they visit or when you meet, it may be at night when clinics are closed so you can’t test her and you can’t get a condom and yet you are not sure of who she has been with while you were away so, in such incidences, PrEP can save you.”* (Participant 4, HIV RDT) | |
| Frequent unplanned sexual encounters | *“My life is very important, and I need to protect it. Besides anything can happen at any time, you know as human beings we catch sexual feelings anytime most especially when we see beautiful women. So, when feelings come sometimes you stop thinking and just end up having unprotected sex, so it is better to take PrEP so that in case anything sexual happens then I am safe.”* (Participant 15, HIVST) | |
| Absence of other HIV preventive measures | *“Now you see, for example, I was transferred from Hoima and taken to Kanungu in a remote area, it is a village. On this side there are no condoms, the government health centers don’t have condoms yet as a man I enjoy sex and my wife is back at home, so if I can have my PrEP with me here, then I can live a happy life as a man having good sex even in the absence of condoms I don’t have to worry about HIV.”* (Participant 15, HIV RDT) | |
| Risky behavior | *“You know us men sometimes we are not faithful to our wives because in most cases we don’t move with them to places where we are transferred. Some of us have many women we are having sex with, I know my friend has had sex with so many girls, yet he doesn’t use condoms. So, if someone is taking their PrEP then they can be protected from HIV even when they sleep with so many women.”* (Participant 30, HIVST) | |
| Condom use errors | *“PrEP is very good because sometimes some men don’t know how to use condoms and end up still getting HIV because they don’t know how to put them on properly and even sometimes condoms burst like when it’s very small or it just bursts just like that. If you are not on PrEP then you can get infected, but if you are on PrEP then you are protected.”* (Participant 8, HIV RDT) | |
| Correct information and positive experiences from peers | *“If I get the right information from the health worker and also if I can get experience from those people who have taken PrEP before and they tell me how it treated them, if they didn’t get side effects then I can take it because then I am sure of what it can do and I can get counseling from my friends that have taken it in case I get any side effects.”* (Participant 20, HIVST) | |
| Information from HIV service providers | *"The truth is this is the first time I am hearing about PrEP; I had never heard of it anywhere else yet it’s a good thing that I can take to protect myself from getting HIV. I think if you the health workers can get this information about PrEP out there then many people like me who didn’t have any information about it can make the right decision and use it.”* (Participant 27, HIV RDT) | |
| Availability | *“If PrEP is made readily available in the lowest health facilities countrywide then me personally, I can take it. The only problem like last time they brought PrEP at the office and some people got but then they were transferred and where they are they can’t access it. But if it is made readily available then many of us can take it up because it’s a good HIV preventive measure. If they bring it to the workplace, that will be better”. Also, when you go to the hospital, they should separate the line for PrEP and ARVs otherwise people will think you are lining for HIV medicine.”* (Participant 11, HIVST) | |
| Ability to sexually engage with multiple sexual partners | *“If PrEP prevents one from getting HIV as you say, then it is a better way because one can have live sex with whoever they want when they want and still be safe and also protect their partners from getting HIV.”* (Participant 4, HIVST) | |
| Liberty to have condomless sex | *“What has been limiting many of us from enjoying sex is the fear of getting HIV and yet we also didn’t want to use condoms because condoms just take away the pleasure, but now that PrEP is here, one can freely enjoy sex without condoms and be safe from HIV. Where can I go to start PrEP?”* (Participant 21,HIV RDT) | |
