# Supplementary material for: Barriers and facilitators to oral PrEP uptake among high-risk men after HIV testing at workplaces in Uganda: a qualitative study
Source: BMC Public Health. 2023 Feb 20;23:365. doi: 10.1186/s12889-023-15260-3 (PMC9940677; doi:10.1186/s12889-023-15260-3)
Supplement: Supplementary file 2 — Supplementary Material 2 [file 12889_2023_15260_MOESM2_ESM.docx]

**Supplementary table 2: Subcategories and narrative quotes for barriers to PrEP uptake**

| Sub-Category | Narrative quotes |
| --- | --- |
| Lack of existing PrEP infrastructure at lowest health facilities | *“While I was in Hoima, I was initiated on PrEP by the team from Hoima hospital but at my workplace, I was transferred to another location that is very far from Hoima, the health facilities around don’t offer any HIV preventive services, I can’t access PrEP because it is not available here so I am stuck and can’t continue with my dose because I can’t refill.”* (Participant 9, HIV RDT) |
| Frequent workplace transfers | *“Now you see with the way we are transferred from station to station from one district to another it is hard to access PrEP because our bosses don’t give you enough time to prepare for the transfer so you can’t go to the hospital where you have been picking the PrEP from to ask for transfer forms and you are not sure whether where you are going you will get a health facility that offers PrEP so it comes hard to keep taking PrEP.”* (Participant 3, HIV RDT) |
| Negative experiences from peers | *Some of my friends have taken PEP before and I have been told that the side effects of ARV drugs are so bad. My friends told me that they had a bad experience so I can’t risk going through what my friends experienced.* (Participant 27, HIVST) |
| Overstated side effects | *“Someone who was taking PrEP told me it makes the body so weak so that you can’t do anything with your body and another one told me that PrEP reduces “manpower”*[erectile dysfunction] *so I can’t risk my body and health taking PrEP. And that in case you have other underlying conditions like hypertension or diabetes this medication makes it worse.”* (Participant 17, HIV RDT) |
| Lack of awareness about PrEP | *“This is the first time I am hearing about this drug called PrEP, I don’t have much information about it for now for me to use it, I need complete knowledge about it, its advantages, disadvantages, side effects before I decide to use it.”* (Participant 13, HIVST) |
| Limited knowledge about prep services | *“PrEP seems a good idea but where can one get such services because I have been in and out of many health facilities and no one has ever told me about that medicine.”* (Participant 22, HIV RDT) |
| Misconception about prep pharmacology | *“One time I went to the hospital, and I was given PEP after being exposed to HIV through an accident. Since PrEP is also a form of ARV then PEP should be enough to protect me from HIV since my body has used ARV before. But I also heard that that drug helps in cleaning the blood from all these other infections and I want to be healthy.”* (Participant 9, HIVST) |
| Preference for injectable medicine | *“I fear taking tablets now you can imagine with PrEP, I have to take them every day as for me I rarely fall sick, so it is so burdening to take tablets every day. I feel it would be better if it were an injection that you can get once like in 3 months so that you know you feel the pain once and are protected for a long period. You don’t know how burdensome it is to take tablets daily*.” (Participant 23, HIVST) |
| Poor adherence | *“It is very hard to take PrEP tablets every day, I will forget because I am a busy person yet for PrEP to be effective it has to be taken every day. But also imagine taking PrEP for a whole month without having sex that means you are taking it for nothing. I can’t manage maybe if one takes PrEP only for the days, they expect to have sex then it would be fine but taking it for long for nothing, no I can’t.”* (Participant 3, HIVST) |
| Perceived poor persistence to medication | *“Taking PrEP is like taking it for life, I can’t manage taking medication for life because you reach a point and just get tired. For people living with HIV at least, they have what is pushing them to take that is they are infected but for HIV negative people I don’t think I can keep up with the daily medication for something I don’t even have.”* (Participant 18, HIVST) |
| The social stigma surrounding PrEP users | *“ARVs are taken by HIV positive people who are sick, but I am not and even them they don’t enjoy so why would I who is HIV negative punish myself by raking PrEP. I only take medicine when I am sick why would I even think of taking ARV when am not infected.”* (Participant 14, HIV RDT) |
| Preference for other HIV preventive campaigns | *“How sure am I that these tablets are effective in HIV prevention? I don't trust it. The option of condoms is better because it creates a barrier between the two people, and you are sure there is no contact. The only option is to leave women alone or use condoms and besides I am young and still abstaining, I am not having sex, so I don’t need to take PrEP now. Maybe when I start having sex then I will think about it then.”* (Participant 24, HIV RDT) |
| Skepticism about PrEP effectiveness | *“There is no way you can assure me that when I take PrEP, I won’t get infected with HIV. I don’t trust the medication because this virus is easy to get once you have sex and there is contact with the other person, so does the medicine prevent contact? Of course not, so there’s no way this medicine you call PrEP can prevent one from getting HIV.”* (Participant 6, HIV RDT) |
| Role of gender and masculinity | *“I need to consult my madam first, I have to talk to her first because if she sees me just taking medicine, she might think that I am already sick with HIV or I am having sex outside marriage and cheating on her, so these are not personal decisions, I have to consult or at least talk to my wife about it to avoid misunderstandings.”* (Participant 19, HIV RDT) |
| Fear of engagement with multiple sexual partners | *“When people know that they can have sex without condoms and they don’t get AIDS, you will not even manage the bad sexual behaviors even in schools and it will increase the other STIs. People will get so many girlfriends, they get tempted to get every beautiful girl and lose the moral culture because after all, they can’t contract the virus.”* (Participant 11, HIV RDT) |
| Perceived low risk for HIV | *“Me, my body is strong. I don’t fall sick just like that, I have even never been injected so I think it is not easy to get HIV anyhow and you see HIV is for promiscuous people, if you ‘tame’ your feelings and eat well caring for your body, it will be strong enough to fight better than ARVs.”* (Participant 6, HIVST) |
